# Assessment of the quality and quantity of naturally induced antibody responses to EBA175RIII–V in Ghanaian children living in two communities with varying malaria transmission patterns

**DOI:** 10.1186/s12936-017-2167-3

**Published:** 2018-01-08

**Authors:** Hamza B. Abagna, Festus K. Acquah, Ruth Okonu, Nii A. Aryee, Michael Theisen, Linda E. Amoah

**Affiliations:** 1 Noguchi Memorial Institute for Medical Research, University of Ghana, Accra, Ghana; 2 Department of Medical Biochemistry, University of Ghana, Accra, Ghana; 3 Department for Congenital Disorders, Statens Serum Institut, Copenhagen, Denmark; 4 Centre for Medical Parasitology at Department of International Health, Immunology and Microbiology, University of Copenhagen, Copenhagen, Denmark

**Keywords:** Malaria, *Plasmodium falciparum*, ELISA, Asymptomatic, Antibodies, Avidity, Transmission

## Abstract

**Background:**

Recent global reports on malaria suggest significant decrease in disease severity and an increase in control interventions in many malaria endemic countries, including Ghana. However, a major driving force sustaining malaria transmission in recent times is the asymptomatic carriage of malaria parasites, which can enhance immune responses against parasite antigens. This study determined the prevalence and relative avidities of naturally induced antibodies to EBA175RIII–V_Ll_ in asymptomatic children living in two communities with varying malaria transmission patterns.

**Methods:**

An asexual stage *Plasmodium falciparum* antigen, EBA175RIII–V_Ll_ was expressed in *Lactococcus lactis*, purified and used in indirect ELISA to measure total and cytophilic IgG concentrations and avidities in children aged between 6 and 12 years. The children were selected from Obom and Abura, communities with perennial and seasonal malaria transmission, respectively. Venous blood samples were collected in July and October 2015 and again in January 2016. The multiplicity of infection and the genetic diversity of EBA175RIII circulating in both sites were also assessed using polymerase chain reaction.

**Results:**

Asymptomatic parasite carriage in the children from Obom decreased from July (peak season), through October and January, however parasite carriage in children from Abura was bimodal, with the lowest prevalence estimated in October. Antibody concentrations over the course of the study remained stable within each study site however, children living in Obom had significantly higher EBA175RIII–V_Ll_ antibody concentrations than children living in Abura (P < 0.05, Mann–Whitney test). Over the course of the study, the relative antibody avidities of EBA175RIII–V_Ll_ IgG antibodies were similar within and between the sites.

**Conclusion:**

Naturally acquired IgG concentrations but not relative antibody avidities to EBA175RIII–V were significantly higher in Obom where malaria transmission is perennial than in Abura, where malaria transmission is seasonal.

**Electronic supplementary material:**

The online version of this article (10.1186/s12936-017-2167-3) contains supplementary material, which is available to authorized users.

## Background

Morbidity and mortality rates associated with malaria in sub-Saharan Africa although on the decline over the past few years, are still very high and a major public health concern [1]. Exposure to infection by *Plasmodium falciparum*, the major causative parasite for malaria in sub-Saharan Africa, results in the development of anti-disease immunity [1] as well as non-sterile anti-parasite immunity, which can protect against high parasitaemia [2]. Maturity of immunity to malaria enables the host to harbour previously encountered *P. falciparum* parasite clones without showing signs of malaria [3–5]. Asymptomatic parasite carriage can be either microscopic or submicroscopic and may persist for extended periods [6, 7] and serve as reservoirs for malaria transmission as no treatment is sought [8].

Antibodies developed against a number of asexual *P. falciparum* sporozoite and merozoite antigens have been determined to be protective in humans [9, 10]. The erythrocyte binding antigen 175 (EBA 175, Pf3D7_0731500) is a *P. falciparum* merozoite ligand that is implicated in erythrocyte invasion, as it is used by the parasite to bind to erythrocytes through interactions with the sialic acid residues of glycophorin A on the erythrocyte during merozoite invasion [11, 12]. EBA175 consists of 6 extracellular regions, R1 through RVI [13]. RII, which consists of two cysteine rich Duffy binding domains, F1 and F2 which have been identified as essential for receptor binding [12] has been produced as a recombinant antigen in *Escherichia coli* [14], Baculovirus [15] and *Pichia pastoris* [16]. Naturally acquired antibody responses against recombinant RII have been suggested to inhibit the merozoite binding to glycophorin A, inhibit invasion in vitro and provide protection against clinical malaria [11, 12, 16–20]. RIII–IV comprises of a dimorphic region, RIII, which is used to distinguish between the FCR3 ‘F’ and CAMP ‘C’ parasite variants [13] as well as the highly conserved RIV and RV regions [7]. EBA175RIII–V has been expressed in *E. coli* [11, 17, 21] and naturally-induced antibodies against the RIII–V region [20, 21] have been suggested to offer protection against disease in some studies [17, 22]. Antibodies against region IV–V of EBA 175 have been shown to have strain transcending growth inhibitory properties, which are more potent than antibodies against the RII region [23] and more significantly correlated with protection against clinical malaria in children [24]. A recent report suggests that antibodies against RIII–V offer similar levels of protection as a 200-fold excess of antibodies against RII [25], suggesting antibodies against RIII–V are more potent than antibodies against RII.

Exposure to malaria parasites in perennial transmission settings is expected to be higher than in seasonal transmission settings. Repeated exposure to malaria antigens is expected to increase antibody affinity and result in an increase in antibody avidity [26], however, some reports on avidity of malaria antibodies have suggested otherwise. One report has suggested that increased exposure to *P. falciparum* parasites did not result in an increase in the relative antibody avidity to merozoite antigens including MSP1 and MSP3 [26]. An earlier study on Gambian children also failed to find an association between asymptomatic *P. falciparum* carriage and antibody avidity [27] despite the fact that asymptomatic infections repeatedly expose the host to the parasite, which should boost immune responses and enhance affinity maturation. These studies put together suggest that more studies are needed to understand the functions of anti-EBA175RIII–V antibodies as well as understand how antibody concentrations and avidity influence asymptomatic *P. falciparum* carriage.

In this study, a new recombinant linear fragment of EBA175RIII–V, EBA175RIII–V_Ll_ was produced in *Lactococcus lactis* and used to monitor changes in the quality and quantity of naturally acquired anti-EBA175 antibodies in school children aged between 6 and 12 years, living in two communities of varying malaria parasite prevalence in southern Ghana.

## Methods

### Study site and population

**Fig. 1:**
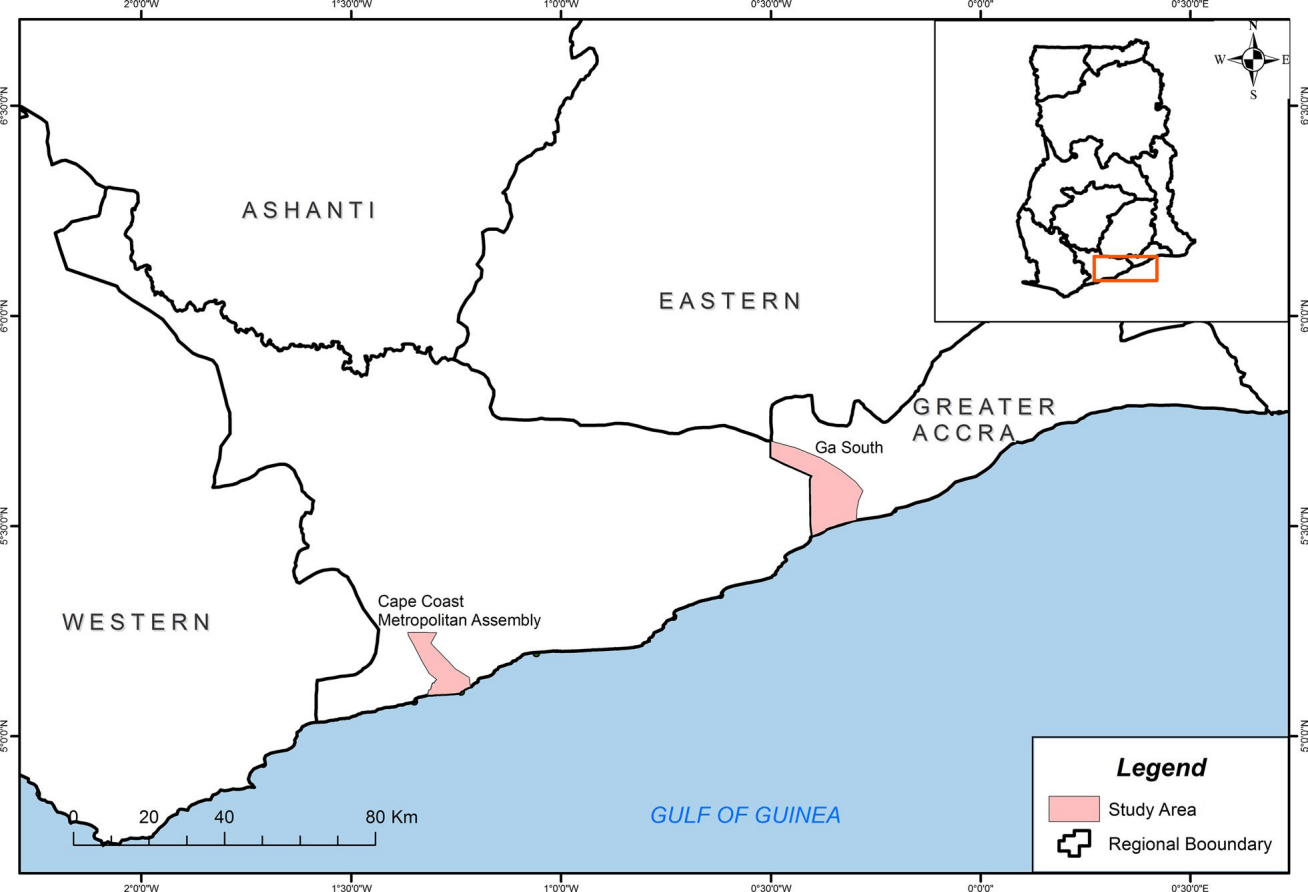
Map of Ghana projecting the location of the study sites Obom and Abura in southern Ghana

A multiple cross-sectional study was conducted on a cohort of 137 children aged between 6 and 12 years. The children were recruited from two public primary schools, one in Obom, in the Obom Domeabra constituency of the Greater Accra Region, and the other in Abura in the Cape Coast metropolis of the Central Region (Fig. 1), both within the coastal savanna zone of Ghana. The peak malaria season in both sites is between July and August but malaria transmission in Obom is perennial and seasonal in Abura [28]. None of the children who were sampled exhibited any signs of clinical malaria.

### Sample collection and processing

The children recruited in this study were part of a larger study where children were recruited into a multiple cross-sectional study that began in February 2015 and ended in January 2016. A total of 64 children from Obom and 73 children from Abura who were present during the July 2015 (peak malaria season), October 2015 (end of peak season) and January 2016 (off peak season) sampling visits were selected for this study. During each collection, 5 ml of venous blood was collected into acid citrate dextrose (ACD) vacutainer tubes and used to prepare thin and thick blood smears (for species identification and parasite density estimation, respectively) as well as to spot filter paper. The filter paper samples were air-dried and stored desiccated at room temperature for DNA extraction within a week of the collection. Plasma was separated by centrifugation from the whole blood and stored frozen at − 20 °C until required.

### Estimation of parasite density

Thick and thin blood smears were processed and observed under ×100 oil immersion using a light microscope [29]. The blood smears were subsequently read by two independent microscopists. The number of infected erythrocytes was counted against 200 white blood cells (WBCs) and then multiplied by 40, based on the assumption that 1 μl of blood contains 8000 WBCs to yield the parasite density [30]. *Plasmodium* species identification was based on morphological differences between species.

### Parasite genotyping

#### Extraction of genomic DNA

DNA extraction was carried out using the Chelex extraction method as previously described [31] with minor modifications. Briefly, two discs of the dried filter paper blood-spots (DBS) were punched using a 3 mm paper punch into a 1.5 ml microcentrifuge tube containing 1120 μl of 0.5% saponin (0.5 g saponin (Sigma-Aldrich, USA) in 100 ml phosphate buffered saline (Sigma-Aldrich, USA), pH 7.4 (PBS) solution. The tubes were vortexed and then left shaking on a shaking incubator overnight at room temperature. The saponin solution was subsequently decanted, the discs washed twice with 1 ml of ice-cold PBS and centrifuged for 10 min at 10,000×*g*. A 150 μl aliquot of 6% Chelex-100 (Sigma-Aldrich, USA) in DNase/RNase-free water was added to each disc. After a 5-min incubation at 95 °C the tubes were centrifuged at 14,000×*g* and the DNase/RNase-free water containing the extracted genomic DNA carefully pipetted into a labelled tube and stored at − 20 °C until further use.

#### Molecular characterization of *P. falciparum* parasites

Amplification of the 18sRNA from *P. falciparum* [32] was used to determine the prevalence of asymptomatic infection in children in each site during all the three sample collection time points. Briefly, the primary PCR reaction consisted of 5 μl of genomic DNA (gDNA), 200 nM dNTPs, 2 mM MgCl_2_, 133 nM each of forward (rPLU6) and reverse (rPLU5) primers (Additional file 1) and 1 U OneTaq DNA polymerase (New England Biolabs, UK). The nested PCR consisted of a similar reaction mix as the primary reaction, except that the primers were replaced with rFal1 (forward) and rFal2 (reverse) and 2 μl of the primary product was used as the template. The positive control used for the speciation reaction was gDNA from the 3D7 strain of *P. falciparum* (MRA 102G).

The RIII domain of EBA175 (EBA175RIII) was amplified from DNA extracted from samples collected in July and October using semi nested PCR with primers listed in Additional file 1. Briefly, 4 μl of genomic DNA (gDNA) was amplified using a PCR reaction mixture that consisted of 200 nM dNTPs, 2 mM MgCl_2_, 133 nM each of forward and reverse primers (R3F and R5R for the primary reaction and R3F and R3R for the secondary reaction), and 0.5 units of One Taq DNA polymerase. PCR products were separated on 2% ethidium bromide-stained agarose gels and visualized under UV illumination. Genomic DNA for *P. falciparum* strains K1 (MRA 159G) and 3D7 (MRA 102G), obtained from MR4, were used as positive controls for the PCR genotyping reactions.

#### Multiplicity of *Plasmodium falciparum* parasite infection

The *merozoite surface protein 1* (*msp1*) and *merozoite surface protein 2* (*msp2*) genes were amplified from the extracted DNA from samples collected in July using a similar process to that described in an earlier study [33]. Briefly, the highly polymorphic block 2 region of *msp*1 and block 3 of *msp*2 were amplified using family specific nested polymerase chain reaction (PCR). The primary 15 μl reaction contained ~ 10–50 ng of extracted DNA, 0.2 mM dNTP mix, 2 mM MgCl_2_, 0.2 mM each of a combination of forward and reverse primers for both *msp*1 and *msp*2 and 0.5 units of OneTaq Polymerase. The family specific *msp*1 and *msp*2 secondary reactions mixture were similar to the primary, however, 1 μl of the primary PCR product was used as template and family specific primers (Additional file 1) were used. A negative control (no template) and positive controls for each allelic family (*msp*1: MRA-159G for KI, MRA-155G for MAD20 and MRA-200G for the RO33, *msp*2: MRA-102G for 3D7 and MRA-155G for FC27) were included in the PCR reactions.

#### Production of EBA175RIII–V_Ll_

The EBA175RIII–V fragment (2280–3900 bp) was amplified from gDNA extracted from the 3D7 *P. falciparum* parasite strain (‘F’ allele) and produced in *L. lactis* using the same expression vector and host as previously described for the production of Pfs230_LlC0_ [34] and primers listed in Additional file 1. Briefly, the EBA 175 RIII–V gene was amplified from gDNA extracted from the 3D7 strain of *P. falciparum* using the 3RF and the R5R primers listed in Additional file 1. The PCR amplicon was subsequently treated with *Bgl*II and *Bam*HI and purified using the DNA Clean & Concentrator™-25 kit (Zymo). The vector, pLEA2 [35] was digested with *Bgl*II, dephosphorylated using alkaline phosphatase and purified from a 1% agarose gel. The vector and fragment were ligated using T4 DNA ligase, transformed into max efficiency DH5α competent cells according to manufacturer’s instructions and plated on LB plates containing 250 μg/ml erythromycin. Colonies containing the EBA175RIII–V plasmid were grown in selective media and plasmids isolated using the EZNA plasmid purification kit (Omega Biotek, USA) according to manufacturer’s protocol. The plasmids were sent for commercial sequencing (Integrated DNA Technology, GE).

#### Expression of EBA175-RIII–V_Ll_

A procedure similar to that previously described for the production of Pfs230_LlC0_ [34] was used. Briefly, the pLEA2.EBA175RIII–V plasmid was transformed into competent *L. lactis* strain MG1363 using the Gene Pulser (BIORAD, USA). Transformed cells were then plated on Yeast Peptone Dextrose media (YPD) plates containing 5 µg/ml erythromycin and then incubated at 30 °C. Transformed *L. lactis* colonies were screened for protein expression by inoculating a colony into 5 ml of YPD media supplemented with 10 µg/µl erythromycin overnight. The culture was subsequently harvested via centrifugation at 4500×*g* for 10 min and the supernatant subjected to SDS-PAGE analysis.

Large scale production of EBA175RIII–V_Ll_ was prepared by inoculating a biofermentor [35, 36] containing 1 L of YPD supplemented with 10 µg/µl erythromycin and set at 30 °C and pH 6.5 with a clone of EBA175RIII-V. After 4–5 h of stationary phase growth (~ 14 h of total growth), the culture was harvested by centrifugation at 10,000×*g* for 20 min.

### Protein purification and characterization

The culture supernatant was clarified by filtration through a 20 µm filter and then purified by affinity chromatography (AC) over a 5 ml HisTrap Ni–NTA column (GE healthcare, USA) using an AKTAxpress purification system (GE healthcare, USA) with wash/equilibration buffer (50 mM sodium phosphate pH 7.0/250 mM sodium chloride/20 mM imidazole) and elution buffer (wash buffer supplemented with 200 mM imidazole).

A 15 µl aliquot of the clarified culture supernatant as well as 15 µl of a tenfold diluted affinity purified protein were subjected to SDS-PAGE analysis as previously described [34]. The gels were either stained with Coomassie blue or subjected to western blot analysis. The western blot was probed with penta-His mouse IgG1 monoclonal antibodies (Thermo Scientific, USA) followed by alkaline phosphatase conjugated goat anti mouse IgG (H + L) secondary antibodies (Thermo Scientific, USA). The blot was finally developed using 1-step nitro-blue tetrazolium and 5-bromo-4-chloro-3′-indolyphosphate (NBT/BCIP) substrate solution (Thermo Scientific, USA).

### Enzyme-linked immunosorbent assay (ELISA)

Enzyme linked immunosorbent assays were performed using a protocol similar to that previously reported by Acquah et al. [34]. Briefly, 100 µl of purified protein EBA175-RIII–V_Ll_ diluted to 1 µg/ml in PBS was coated onto NUNC maxisorp™ ELISA plates overnight at 4 °C. Plates were washed four times using PBS supplemented with 0.05% Tween 20 (PBS/T) and blocked with 3% (w/v) skimmed milk powder (Marvel, UK) in PBS/T. Recombinant polyclonal human IgG (PB055) was used as a standard, at a starting concentration of 1000 ng/ml (100 µl) and serially diluted threefold for 7 additional concentrations. The plates were then incubated for an hour with 100 µl/well of test plasma or a negative control (malaria naïve) sample consisting of a pool of malaria naïve plasma all diluted 200-fold at room temperature, and then washed four times with PBS/T. The plates were subsequently incubated with goat anti-human IgG-HRP secondary antibodies (Invitrogen, USA). All plates were developed by adding 50 µl of 3,3′,5,5′-tetramethylbenzidine (TMB) solution for 15 min and then stopped with 50 µl of 2 M H_2_SO_4_. Absorbance of coloured products of the reaction was measured immediately after stopping the reactions at a wavelength of 450 nm.

### IgG1 and IgG3 ELISA

A procedure similar to that described above for IgG was used with some minor modifications. Basically, the test samples were diluted 1:50 and the standard used was a pool of plasma previously identified as having high IgG1 and IgG3 concentrations at a starting concentration of 1:50 and diluted twofold for 6 extra dilutions. The plates were processed as above. The temperature setting for the 1 h sample incubation was increased to 37 °C and the secondary antibodies used were goat anti-human IgG1 and IgG3, respectively.

### Relative avidity ELISA

A procedure similar to the total IgG ELISA described above was used, however the plasma samples after the 1 h incubation were washed three times and then incubated with 2.4 M sodium thiocyanate (NaSCN) for 10 min and then subsequently washed off thrice with PBS/T prior to the addition of the secondary antibody [26, 37]. Avidity for the subclass proceeded similar to the subclass ELISA with the added NaSCN step.

### Data analysis

The physical and chemical properties of the antigen were obtained using ProtParam [38]. ELISA data was entered into Excel, converted to antibody concentrations using ADAMSEL (Ed Remarque) and analysed using GraphPad Prism v5 and the R statistical software (Version 3.4.0). The frequency and other column statistics including T-tests and One way ANOVA (Kruskal–Wallis) tests were determined using GraphPad Prism v5. A non-parametric correlation (Spearman, GraphPad Prism) was used to assess correlation between age and antibody concentration and antibody avidity.

The R statistical software (Version 3.4.0) was used for the assessment of antibodies against EBA175. To project asymptomatic parasite carriage during the subsequent sampling time point in the school children, a mixed effects regression model for repeated measurements was fitted to antigen-specific antibody data using the R package lme4 [39]. Initially, antigen-specific antibody data and relative avidity data were log2 transformed and entered into the model as fixed effects with age as an interaction term, whilst visit time point and town were entered as random effects. This model was compared to other models that dropped the age interaction term or one of the random effects, and the best model, based on the lowest AIC values was selected for further analysis. The best model had log2 transformed antibody level and log2 relative avidity index (RAI) transformed as the fixed effect and study town as a random effect since the interaction term (age) and visit time point did not significantly contribute to the models. P-values for the intercept and fixed effect were obtained by likelihood ratio tests (with Laplace Approximation) (Table 3).

The relative avidity index (RAI) for an antibody was calculated as the ratio of the mean IgG concentration of the sodium thiocyanate-treated sample to the mean IgG concentration of the untreated sample multiplied by 100 (Avidity index = [antibodies following Sodium thiocyanate (NaSCN) treatment/antibodies without NaSCN treatment] × 100). Statistical significance was defined as *P* ≤ 0.05 unless otherwise stated.

Data from healthy (non parasitaemic) children were excluded from all analysis that compared antibody responses (concentration and avidity) between the three-time points.

## Results

The study used samples from 137 school children between 6 and 12 years of age. The number of asymptomatic children detected by the microscopic evaluation of thick blood smears ranged between 10.9 and 71.9% in Obom and 17.8–58.9% in Abura (Table 1) and identified the presence of *Plasmodium malariae* mono infections in four children from Obom in July. Asymptomatic parasite carriage determined by PCR ranged between 59.4 and 68.8% in Obom and 18.9–75.3% in Abura (Table 1). The mean age (standard error of mean, SEM) of the school children was 9.09 (0.215) years in Obom and 8.79 (0.178) years in Abura was not significantly different between the two sites (P > 0.05, Mann–Whitney test). The geometric mean *P. falciparum* parasite density (PD) reduced in moving from July (the peak) through October (the end of the peak) to January (the off peak) season in both sites, with PD in July being significantly different from PD in both October and January in both Obom (P < 0.05, Dunn’s Multiple Comparison Test) and Abura (P < 0.001, Dunn’s Multiple Comparison Test).

**Table 1:** Characterization of study participants

	*P. falciparum* PCR (%)	Microscopy (%)
Abura		
July	75.3	58.9
Oct	18.9	17.8
Jan	52.1	30.1
Obom		
July	68.8	71.9^a^
Oct	67.2	26.6
Jan	59.4	10.9

Data presented from samples collected from 64 and 73 children living in Obom and Abura respectively

^a^Four children in Obom carried *P. malariae* mono infections

### Parasite genotyping

The geometric mean multiplicity of infection (95% confidence interval) determined by genotyping the *msp1* gene for asymptomatic parasite infection in Obom in July was 2.094 (1.873–2.341), which was significantly (P < 0.0001, Mann–Whitney two-tailed test) higher than 1.397 (1.238–1.577) determined in the asymptomatic children living in Abura in the same month.

Agarose gel electrophoresis of the EBA175RIII PCR amplicons yielded fragments that were either ~ 740 bp (‘C’ allele) or ~ 820 bp (‘F’ alleles). The band sizes are different from the 715 and the 795 bp fragments that are characterized by amplification of RIII using the primers EBA1, 2, 3 and 4 [22] due to the use of the antigen cloning primers for the reactions (Additional file 1: Table S1). Parasites identified in almost all of the samples genotyped predominantly belong to a single allelic family, with only one sample from Obom containing parasites belonging to both allelic families. The ‘F’/’C’ allelic ratio ranged from 1.1 in Obom to 1.2 in Abura (Table 2).

**Table 2:** Allelic distribution of EBA175

Allele	Abura (%, N)	Obom (%, N)
F	54 (34)	51.6 (33)
C	46 (29)	46.9 (30)
F + C	0	1.6 (1)

The percentages of the alleles with the exact count in brackets

### Production of recombinant EBA175RIII–V_Ll_

**Fig. 2:**
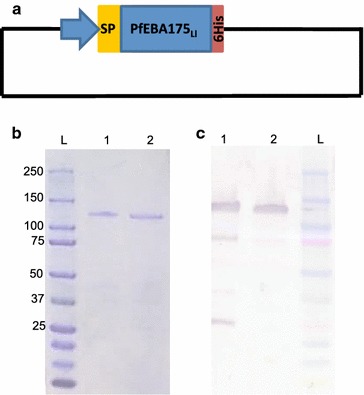
*Plasmodium falciparum* EBA175RIII–V construct and expressed antigen. A schematic representation of the 1620 bp region of EBA175RIII–V cloned into the pLEA2 expression vector, which contains the nucleotide sequence of a hexahistidine tag inframe of the multiple cloning site (**a**). The culture supernatant (1) containing the secreted protein as well as the purified protein (2) was analysed by SDS-PAGE followed by coomassie staining (**b**) and a western blot probed with penta-His mouse monoclonal antibody (IgG1) (**c**)

The 1620 bp region of EBA175 (bp 2280–3900) (Fig. 2a), representing RIII–V was successfully expressed as a secreted protein in *L. lactis* MG1363. Recombinant EBA175RIII–V_Ll_ (amino acid 760–1300 of EBA175) was purified from culture supernatants on a HisTrap FF Crude column (GE Healthcare, DK) yielding a ~ 125 kDa product (Fig. 2b). Purified antigen was used to assess naturally acquired immune responses to EBA 175 and the relative avidity of these antibodies.

### Characterization of naturally acquired antibody responses against EBA175RIII–V_Ll_

**Fig. 3:**
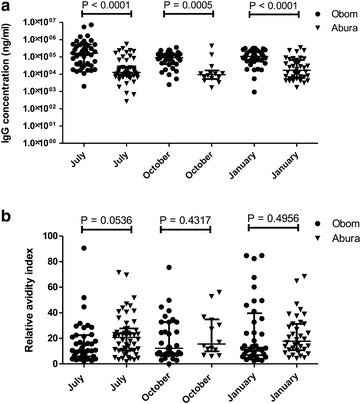
Characterization of IgG responses against EBA175RIII–V_Ll_. Antibody concentrations (ng/ml) of plasma obtained from the enrolled children from Obom and Abura (**a**) diluted 1:200 was determined using indirect ELISA and a EBA175RIII–V_Ll_ as the antigen coated onto the ELISA plate and goat anti-human IgG used as the secondary antibody. The relative avidities of these same plasma samples were determined using a modified indirect ELISA assay where an incubation of the bound plasma samples obtained from children Obom and Abura (**b**) were treated with sodium thiocyanide is incorporated into the protocol. Plasma samples were obtained from whole blood collected from the children during the months of July 2015, October 2015 and February 2016. Data in the graphs are represented as the median with the interquartile range

There was no significant change (P > 0.05, Dunn’s multiple comparative test) in the median concentrations of antibodies against EBA175RIII–V_Ll_ over the course of the changing malaria seasons in asymptomatic children from both Obom and Abura (Fig. 3a). However, antibody responses measured at each time point in Obom were significantly higher [P < 0.0001 (July), 0.0005 (October) and 0.0001 (January), Mann–Whitney test] than those measured in Abura. The general decrease in antibody concentrations observed in moving from July (the peak) to October (off-peak season) in Obom was not apparent in Abura (Fig. 3a). The trends in IgG responses to EBA175RIII–V_Ll_ in the children were similar to those against the non-repetitive domain of *P. falciparum* Glutamate Rich Protein (GLURP.RO) (Additional file 2). The relative avidity indices of antibodies measured in Obom were generally lower than in Abura (Fig. 3b), however, no significant difference was identified in the median RAI of IgG in July, October and January in either Abura or Obom (P > 0.05, Dunn’s multiple comparative test).

**Fig. 4:**
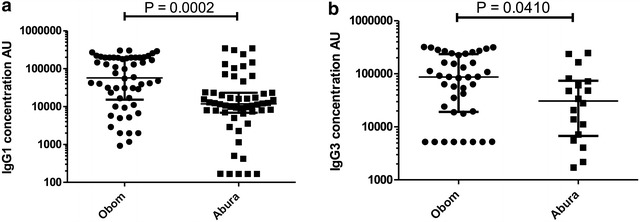
Characterization of cytophilic antibody responses. IgG1 (**a**) and IgG3 (**b**) antibody concentrations to EBA175RIII–V_Ll_ in asymptomatic children from Obom and Abura measured in the peak malaria season (July). Processes similar to that described in Fig. 3 were used to determine the concentrations and avidity of IgG1 and IgG3, the only difference was that goat anti-human IgG1 and goat anti-human IgG3 secondary antibodies were used in place of the goat anti-human IgG. The graphs represent the median antibody concentrations with interquartile range as error bars

The levels of IgG1 and IgG3 antibodies were significantly higher in asymptomatic children from Obom compared to the asymptomatic children from Abura (Mann–Whitney test, P = 0.0002 and 0.0410, respectively) (Fig. 4a, b).

The concentration of IgG antibodies to EBA175RIII–V_Ll_ in asymptomatic children from both Obom and Abura did not correlate significantly with age at any time point (P > 0.05, Spearman r = 0.3032, 0.2171 and 0.1597 in Obom and − 0.04814, 0.3033 and 0.04804 in Abura for July, October and January, respectively). Fewer children with high antibody avidities in July and October harboured parasites in the subsequent visit, October and January, respectively (Table 3).

**Table 3:** Influence of antibody concentration and relative avidity index (RAI) on the future carriage of *P. falciparum* parasites in different malaria transmission settings

Fixed effects	Estimate std	Error	z value	Pr (> |z|)
(Intercept)	0.4623	3.9658	0.117	0.9072
log2(Conc)	0.1397	0.1345	1.038	0.299
log2(RAI)	0.5251	0.2379	2.207	0.0273*

*Conc.* antibody concentrations in ng/ml, *RAI* relative avidity index

* P < 0.05

## Discussion

The early identification of the function RII and protective properties of antibodies against EBA175RII led to it gaining vaccine candidate status several years ago [18, 40]. The function of RIII–V has still not been confirmed [25] and the functions of antibodies against RIII–V of EBA175 have not been as extensively characterized as RII most likely due to the dimorphic nature of RIII. EBA175 RII was earlier classified as highly conserved [41], although some studies have identified RII to be highly polymorphic [42, 43] and anti-EBA175R2 antibodies not to be associated with protection [44, 45]. Naturally induced antibodies against *E. coli* produced EBA175RIII–V has been found to be protective [25, 46] and mice anti-EBA175RIII–V suggested to exhibit strain-transcending activity [23]. However, more studies are needed to characterize the functions of antibodies against EBA175RIII–V.

In this study, a new recombinant EBA175RIII–V_Ll_ was produced and used to evaluate changes in the quantity and quality of naturally induced antibody responses developed against EBA175RIII–V in asymptomatic children living in communities with varying malaria transmission intensity across changing malaria seasons.

The recombinant antigen was produced in high yields, similar to that of Pfs230C0_*Ll*_ [34] and purified using a similar one-step purification process as Pfs230C0_*Ll*_ [34]. The antigen migrated as a ~ 125 kDa protein on a polyacrylamide (PAGE) gel (Fig. 2b, c), although the theoretically calculated size of the protein is ~ 60 kDa. A similarly large size antigen was produced when EBA175RIII–V was expressed in *E. coli* [23]. This migratory pattern could be a result of the antigen having a high negative charge and a grand average of hydropathicity (GRAVY) score of − 1.384. Proteins with low GRAVY scores are noted to migrate slower than expected on SDS-PAGE gels [47]. A similar phenomenon was observed after the production and purification of PfGLURP in *L. lactis*. PfGLURP is a negatively charged protein, which lacks tryptophan residues and migrated at ~ 92 kDa although the theoretical size is ~ 55 kDa [48]. EBA175RIII–V_Ll_ was produced in *L. lactis* due to the similar codon usage between *L. lactis* and *P. falciparum*, and the fact that the fragment did not require disulfide bridge formation. *Lactococcus lactis* has also been used for the successful production of a number of malaria vaccine candidates including GMZ2 [49] and R0.6C [35] and antigens such as Pfs230C0_*Ll*_ [34].

The geometric mean parasite density determined in July was significantly higher than that recorded in October and January in both Obom and Abura although the prevalence of children harbouring asymptomatic *P. falciparum* parasites determined by PCR remained stable over the same period in Obom. The prevalence of *P. malariae* and possibly other *Plasmodium* species could have been higher if more sensitive molecular tools were employed. The prevalence of *P. malariae*, the second most predominant *Plasmodium* species in Ghana has recently been reported to be as high as 12.7% in the tropical rainforest zone of Ghana [50]. The distribution of parasites with different *eba*175 alleles in the asymptomatic children from both Obom and Abura were similar, with an F/C ratio ranging between 1.1 in Obom and 1.2 in Abura (Table 2). Genetic diversity in EBA175RIII–V has been suggested to be limited [23] and the allelic distribution patterns found to be relatively stable over extended periods of time [51, 52]. The genotyping data did not portray the usual high prevalence of ‘F’ allele compared to the ‘C’ as has been previously reported in a number of West African countries including Ghana [22, 51, 53]. However, these previous studies were conducted on symptomatic samples. The geometric mean multiplicity of infection in asymptomatic children in Obom was significantly higher than that estimated for the children living in Abura, most likely due to the higher levels of malaria transmission in Obom compared to Abura.

Naturally-induced antibody responses to EBA175RIII–V_Ll_, a ‘F’ type antigen was high in Obom and low in Abura. The even distribution of the dimorphic EBA175 alleles in both sites likely did not preferentially influence the antibody responses in either site. Naturally induced IgG responses in Obom were significantly (P < 0.001, Mann–Whitney test) higher in Obom than those measured in Abura (Fig. 3a, b) most likely due to increased exposure to parasites by children living in the perennial transmission setting, Obom compared to Abura, the seasonal transmission setting (Table 1a). The median IgG concentrations of EBA175RIII–V_Ll_ measured in children from Obom at all the time points were significantly (P < 0.0001, Mann–Whitney test) higher than those measured in Abura, even in July when the prevalence of asymptomatic parasite carriage estimated by both microscopy and PCR was higher than in Obom. This finding supports earlier studies that used naturally acquired antibody responses to merozoite antigens including the apical membrane protein 1 (AMA1) and the merozoite surface protein 1 (MSP1) to differentiate between communities with different malaria transmission intensities [54–57].

No significant differences were observed in the median IgG concentrations of anti-EBA175RIII–V antibodies across the different malaria seasons in both Obom and Abura. This could be a result of boosting of antibody responses by the presence of infecting parasites in asymptomatic children, whose prevalence was maintained between October and January in both sites. This supports the suggestion that the presence of concurrent parasitaemia has been found to increase antibody responses to EBA175 antigens [24].

The IgG subclass responses against EBA175 have been suggested to be predominantly cytophilic (IgG1 and IgG3) [58, 59]. IgG1 and IgG3 responses to EBA175RIII–V have also been associated with lower parasitaemia and protection from malaria [17, 59, 60]. In this study IgG1 and IgG3 levels in children from Obom in July were higher than those from Abura (Fig. 4), which was not surprising as total IgG levels were also significantly higher in Obom than in Abura.

The relative avidity index (RAI) of IgG antibodies against EBA175RIII–V_Ll_ was significantly inversely associated with parasite carriage during the subsequent quarterly visit (Table 3). The relative avidity of antibodies against EBA175RIII–V_Ll_ in Obom was generally lower, although not significantly different from those measured in Abura (P > 0.05, paired *t* test) (Fig. 3c, d). The low avidity of antibodies in Obom support previous studies that have suggested impaired affinity maturation to malaria antigens in settings with high perennial exposure to malaria parasites [61]. Maturation of humoral immune responses is expected to result in the production of antibodies with increased avidity [62, 63], however, constant parasite carriage in areas with high perennial malaria transmission, such as Obom can result in reduced antibody affinities as has been previously reported [64].

### Limitations

*Plasmodium falciparum* parasites were the only species of malaria parasites characterized by PCR in the study. The children were also not followed actively or passively so could have had additional exposure to *P. falciparum* parasites that were not captured at the sampling time points.

## Conclusion

Naturally acquired IgG concentrations but not relative antibody avidities to EBA175RIII–V were significantly higher in Obom where malaria transmission is perennial than in Abura, where malaria transmission is seasonal.

## Additional files

**Additional file 1.** Primers for cloning *P. falciparum* eba175RIII–V as well as for detecting and genotyping *P. falciparum* parasites.

**Additional file 2.** IgG responses to EBA175 and the Glutamate Rich Protein (GLURP.RO) measured in plasma samples obtained from the school children in October 2015 and January 2016.

## Abbreviations

EBA175: *Plasmodium falciparum* erythrocyte binding antigen 175 (Pf3D7_0731500)
bp: base pair
aa: amino acid
*msp*: merozoite surface protein
ELISA: enzyme linked immunosorbent assay
DBS: dried blood spot
*L. lactis*: *Lactococcus lactis*
NaSCN: sodium thiocyanate
RAI: relative avidity index
