# Supplementary material for: Assessment of the quality and quantity of naturally induced antibody responses to EBA175RIII–V in Ghanaian children living in two communities with varying malaria transmission patterns
Source: Malar J. 2018 Jan 8;17:14. doi: 10.1186/s12936-017-2167-3 (PMC5759240; doi:10.1186/s12936-017-2167-3)
Supplement: Supplementary file 1 — Additional file 1. Primers for cloning P. falciparum eba175RIII–V as well as for detecting and genotyping P. falciparum parasites. [file 12936_2017_2167_MOESM1_ESM.docx]

**Additional file 1**

Primers for cloning *P. falciparum* eba175RIII-V as well as for detecting and genotyping *P. falciparum* parasites.

Primer sets and annealing temperatures for the amplification of *P. falciparum* eba175 RIII-V, **eba**175RIII, *18 SSU rRNA* and the *msp*1 and *msp*2 genes.

| **Gene/primer name** | **Primer sequence** | **Annealing temp** |
| --- | --- | --- |
| ***Eba 175*** |  |  |
| R3F | TAATGGATCCTCAAGATCTCAAGAAGCAGTTCTGAGGA | 70.5 °C |
| R3R | CCATAGATCTTCTCAACATTCATATTAACAATTC | 62.2 °C |
| R5R | CCATAGATCTTGATGCCTAGGAACATTCCCAGAATTTCC | 70.5 °C |
| ***msp* 1** |  |  |
| M1- OF: | CTAGAAGCTTTAGAAGATGCAGTATTG | 54 °C |
| M1- OR: | CTTAAATAGTATTCTAATTCAAGTGGATCA | 54 °C |
| M1- KF: | AAATGAAGAAGAAATTACTACAAAAGGTGC | 59 °C |
| M1- KR: | GCTTGCATCAGCTGGAGGGCTTGCACCAGA | 59 °C |
| M1- MF: | AAATGAAGGAACAAGTGGAACAGCTGTTAC | 59 °C |
| M1- MR: | ATCTGAAGGATTTGTACGTCTTGAATTACC | 59 °C |
| RO33- F: | TAAAGGATGGAGCAAATACTCAAGTTGTTG | 59 °C |
| RO33- R: | CAAGTAATTTTGAACTCATGTTTTAAATCAGCGTA | 59 °C |
| ***msp* 2** |  |  |
| M2- OF: | ATGAAGGTAATTAAAACATTGTCTATTATA | 54 °C |
| M2- OR: | CTTTGTTACCATCGGTACATTCTT | 54 °C |
| S1fw: | GCTTATAATATGAGTATAAGGAGAA | 50 °C |
| 3D7 N5rev: | CTGAAGAGGTACTGGTAGA | 50 °C |
| FC27 M5rev: | GCATTGCCAGAACTTGAA | 50 °C |
| ***18 SSU rRNA*** |  |  |
| rPLU6 | TTA AAA TTG TTG CAG TTA AAA CG | 54 °C |
| rPLU5 | CCT GTT GTT GCC TTA AAC TTC | 59 °C |
| rFAL1 | TTA AAC TGG TTT GGG AAA ACC AAA TAT ATT | 59 °C |
| rFAL2 | ACA CAA TGA ACT CAA TCA TGA CTA CCC GTC | 64°C |
