# Supplementary material for: Assessment of the quality and quantity of naturally induced antibody responses to EBA175RIII–V in Ghanaian children living in two communities with varying malaria transmission patterns
Source: Malar J. 2018 Jan 8;17:14. doi: 10.1186/s12936-017-2167-3 (PMC5759240; doi:10.1186/s12936-017-2167-3)
Supplement: Supplementary file 2 — Additional file 2. IgG responses to EBA175 and the Glutamate Rich Protein (GLURP.RO) measured in plasma samples obtained from the school children in October 2015 and January 2016. [file 12936_2017_2167_MOESM2_ESM.docx]

Additional file 2. IgG responses to EBA175 and the glutamate rich protein (GLURP.RO) measured in plasma samples obtained from the school children in October 2015 and January 2016

Indirect ELISAs were performed on samples collected in October and January substituting EBA175RIII-V_Ll_  for *Pf*GLURP(R0) [1] to compare the trends in antibody responses to EBA175RIII-V and an additional asexual parasite antigen. Data in the graph represent the median antibody concentrations with the interquartile ranges as the error bars. RO, *Pf*GLURP(R0); EBA, EBA175RIII-V_Ll_ .

Statistical analysis of IgG responses against RO and EBA175

| **Dunn's Multiple Comparison Test** | **Difference in rank sum** | **Significant? P < 0.05?** | **Summary** |
| --- | --- | --- | --- |
| **RO** |  |  |  |
| Obom October vs Obom January | -4.161 | No | ns |
| Obom October vs Abura October | 31.77 | Yes | * |
| Obom October vs Abura January | 28.49 | Yes | ** |
| Obom January vs Abura October | 35.93 | Yes | ** |
| Obom January vs Abura January | 32.65 | Yes | *** |
| Abura October vs Abura January | -3.282 | No | ns |
| **EBA** |  |  |  |
| Obom October vs Obom January | -5.079 | No | ns |
| Obom October vs Abura October | 42.5 | Yes | *** |
| Obom October vs Abura January | 38.17 | Yes | *** |
| Obom January vs Abura October | 47.58 | Yes | *** |
| Obom January vs Abura January | 43.25 | Yes | *** |
| Abura October vs Abura January | -4.331 | No | ns |

Antibody responses against RO and EBA175 were similar in October and January within both sites; however, they were significantly different between the sites at all the different time points.

1. Amoah LE, Nuvor SV, Obboh EK, Acquah FK, Asare K, Singh SK, Boampong JN, Theisen M, Williamson KC. Natural antibody responses to Plasmodium falciparum MSP3 and GLURP(R0) antigens are associated with low parasite densities in malaria patients living in the Central Region of Ghana**.** Parasit Vectors. 2017; 10**:**395.
